# Protective Role of GABA in Aromatic Rice Under Lead and Cadmium Toxicity: Physiological and Biochemical Insights

**DOI:** 10.3390/plants14162561

**Published:** 2025-08-17

**Authors:** Umair Ashraf, Shakeel Ahmad Anjum, Fahd Rasul, Muhammad Ansar Farooq, Muhammad Abrar, Farhat Abbas, Chuan Jin, Waseem Hassan, Xiangru Tang, Zaid Khan

**Affiliations:** 1 Department of Botany, Division of Science and Technology, University of Education, Lahore 54770, Punjab, Pakistan; 2 Department of Crop Science and Technology, College of Agriculture, South China Agricultural University, Guangzhou 510642, China; 3 Upper Peninsula Research and Extension Center, Michigan State University, Chatham, MI 49816, USA; 4 Department of Agronomy, University of Agriculture, Faisalabad 38040, Punjab, Pakistan; 5 Institute of Environmental Sciences and Engineering, School of Civil and Environmental Engineering, National University of Sciences and Technology, Islamabad 44000, Pakistan; 6 CFAES Rattan Lal Center for Carbon Management and Sequestration, School of Environment and Natural Resources, The Ohio State University, Columbus, OH 43210, USA; 7 State Key Laboratory of Herbage Improvement and Grassland Agro-ecosystems, College of Ecology, Lanzhou University, Lanzhou 730000, China; 8 Institute of Tropical Fruit Trees, Hainan Academy of Agricultural Sciences/Key Laboratory of Genetic Resources Evaluation and Utilization of Tropical Fruits and Vegetables (Co-Construction by Ministry and Province), Ministry of Agriculture and Rural Affairs/Key Laboratory of Tropical Fruit Tree Biology of Hainan Province, Haikou 571100, China; 9 School of Ecology, Hainan University, Haikou 570228, China; 10 Department of Soil and Environmental Sciences, Muhammad Nawaz Shareef University of Agriculture, Multan 60000, Punjab, Pakistan; 11 College of Architectural Engineering, Shenzhen Polytechnic University, Shenzhen 518055, China

**Keywords:** antioxidants, chlorophyll, heavy metals, photosynthesis, yield, micronutrients

## Abstract

Lead (Pb) and cadmium (Cd) severely impair rice growth, yield, and grain quality. This study assessed the role of exogenous gamma-aminobutyric acid (GABA) in mitigating Pb and Cd toxicity in aromatic rice ‘Guixiangzhan’. Treatments included the control (no Pb, Cd, or GABA), GABA (1 mM), Pb (800 mg/kg of soil)+GABA, Cd (75 mg/kg of soil)+GABA, Pb+Cd+GABA, Pb, Cd, and Pb+Cd without GABA. GABA improved chlorophyll and carotenoid, protein, proline and GABA contents whilst reducing oxidative stress under Pb/Cd toxicity. GABA application regulated antioxidant enzyme activities, net photosynthesis, and gas exchange, while its effects on nitrate reductase and glutamine synthetase were variable. Compared with Pb+Cd, the grain yields were 34.03%, 31.94%, 15.88%, 24.86%, and 17.32% higher in (Pb, Cd, Pb+Cd)+GABA, Pb, and Cd treatments, respectively. Furthermore, GABA reduced Pb and Cd accumulation in aboveground parts, while Ca, Mg, Fe, Cu, Zn, and Mn levels varied across treatments. Cd translocation was more from root-to-leaves, while Pb translocation was more from leaves-to-grains. Grain Pb and Cd positively correlated with their root, stem, and leaf contents but negatively with mineral nutrients. Overall, exogenous GABA mitigated Pb and Cd toxicity in aromatic rice.

## 1. Introduction

The accumulation of heavy metals in agricultural lands due to anthropogenic activities poses a serious threat to safe food production worldwide [1,2]. These toxic metals readily mobilize from soil to plants and accumulate in edible plant parts when soil levels exceed permissible limits [3]. In addition to natural processes, major anthropogenic activities such as industrial effluents, the release of xenobiotic contaminants from factories, and untreated wastewater disposal, are key sources of pollution in agricultural ecosystems [4].

Lead (Pb), one of the most toxic heavy metals, severely impedes plant development by disrupting normal metabolism and plant–nutrient interactions [5]. Previously, Sevik, Ozel [6] reported that Pb uptake, accumulation, and distribution within plant organs depend on the concentration and plant genotype. Moreover, Pb inhibits root–shoot growth, photosynthesis, nutrient uptake, and antioxidant activity and reduces rice yield and quality. Its availability and uptake largely depend on soil physicochemical properties, plant genetic and morphological traits, and Pb concentration in the soil solution [7].

Once cadmium (Cd), a toxic element, enters plants, it disrupts ionic homeostasis, alters source–sink relationships, and affects the antioxidative defense system, ultimately inhibiting growth [8]. Cd generally reduces the enzymatic activities involved in CO_2_ fixation and photosynthetic pigments in plants [9]. Previously, Srivastava, Pandey [10] reported substantial reductions in seedling growth, biomass accumulation, photosynthesis, and photosynthetic pigments, along with alterations in the plant defense system, in rice plants under individual and combined Cd and Pb stress. Both Pb and Cd, as redox-active metals, cause oxidative stress in plants [11], while antioxidants and enzymes involved in the glutathione–ascorbate cycle are largely affected by Pb/Cd stress, leading to a marked reduction in protein thiols [12]. Hence, both Cd and Pb are toxic to plants; however, their effects vary based on exposure levels and the specific plant species or cultivar.

Gamma-aminobutyric acid (GABA) acts as a signaling compound to regulate physio-biochemical responses and the growth of plants under stress conditions [13]. Podlešáková et al. [14] reported that intracellular and/or endogenous GABA production is very low under normal conditions but is enhanced significantly when plants are exposed to stress conditions. The GABA shunt is responsible for both the synthesis and regulation of optimal GABA levels and involves three key reactions catalyzed by the cytosolic enzyme glutamate decarboxylase (GAD) and the mitochondrial enzymes GABA transaminase (GABA-T) and succinic semialdehyde dehydrogenase (SSADH). GAD is a key enzyme involved in GABA metabolism that responds strongly to abiotic stresses and plays a crucial role in enhancing plant stress tolerance via GABA pathways [15,16]. Additionally, GABA promotes the activity of GABA-T and SSADH, supporting the continuity of the GABA shunt and the tricarboxylic acid (TCA) cycle. Both GABA-T and SSADH are notably upregulated under abiotic stress conditions and modulate plant resilience [17]. Generally, GABA levels in plant tissues range from 0.03 to 2.0 µmol g^−1^ fresh weight but increase many fold in response to abiotic stresses [18].

Significant positive effects of various phytohormones, including GABA, on plant development under different stresses have been reported [19]; however, the use of exogenous GABA to alleviate individual or combined Pb/Cd stress in aromatic rice (a special rice group known for its aroma and excellent cooking qualities) has been rarely explored. Therefore, this study aimed to examine the role of GABA application in regulating growth, yield, physio-biochemical responses, and Pb/Cd and mineral nutrient uptake and accumulation in aromatic rice under individual and combined Pb/Cd toxicity

## 2. Results

**Figure 1:**
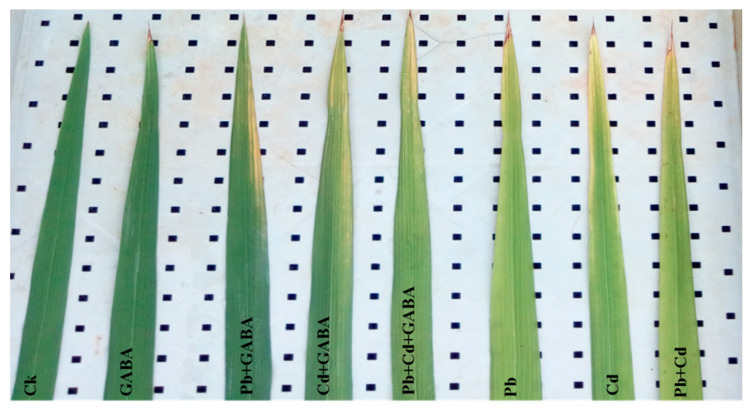
The physical appearance of flag leaves in rice plants under individual and combined Pb and Cd stress, with and without GABA application, showed noticeable differences. Leaves from GABA-treated plants appeared greener and healthier compared to those from non-treated plants under Pb, Cd, and combined Pb+Cd stress, exhibiting signs of chlorosis and stress-induced discoloration. Ck: control (no Pb, Cd, or GABA), GABA (1 mM), Pb (800 mg/kg of soil)+GABA, Cd (75 mg/kg of soil)+GABA, Pb+Cd+GABA, Pb, Cd, and Pb+Cd without GABA.

Visual assessment of rice leaves under different treatments clearly showed the protective effect of GABA against individual and combined Pb/Cd stress. In the control (Ck) and GABA-alone treatments, leaves appeared green and healthy, indicating normal physiological status. In contrast, leaves subjected to Pb, Cd, or combined Pb+Cd stress without GABA showed visible signs of stress, including chlorosis, pale coloration, and reduced leaf greenness whereas the application of GABA under individual (Pb, and Cd) or combined (Pb+Cd) treatments substantially improved leaf appearance, restoring greener pigmentation and reducing visible damage. These observations suggested that exogenous GABA mitigated the adverse visual symptoms of Pb and Cd toxicity in aroma rice (Figure 1).

### 2.1. Malondialdehyde (MDA), Electrolyte Leakage (EL), and H_2_O_2_

**Figure 2:**
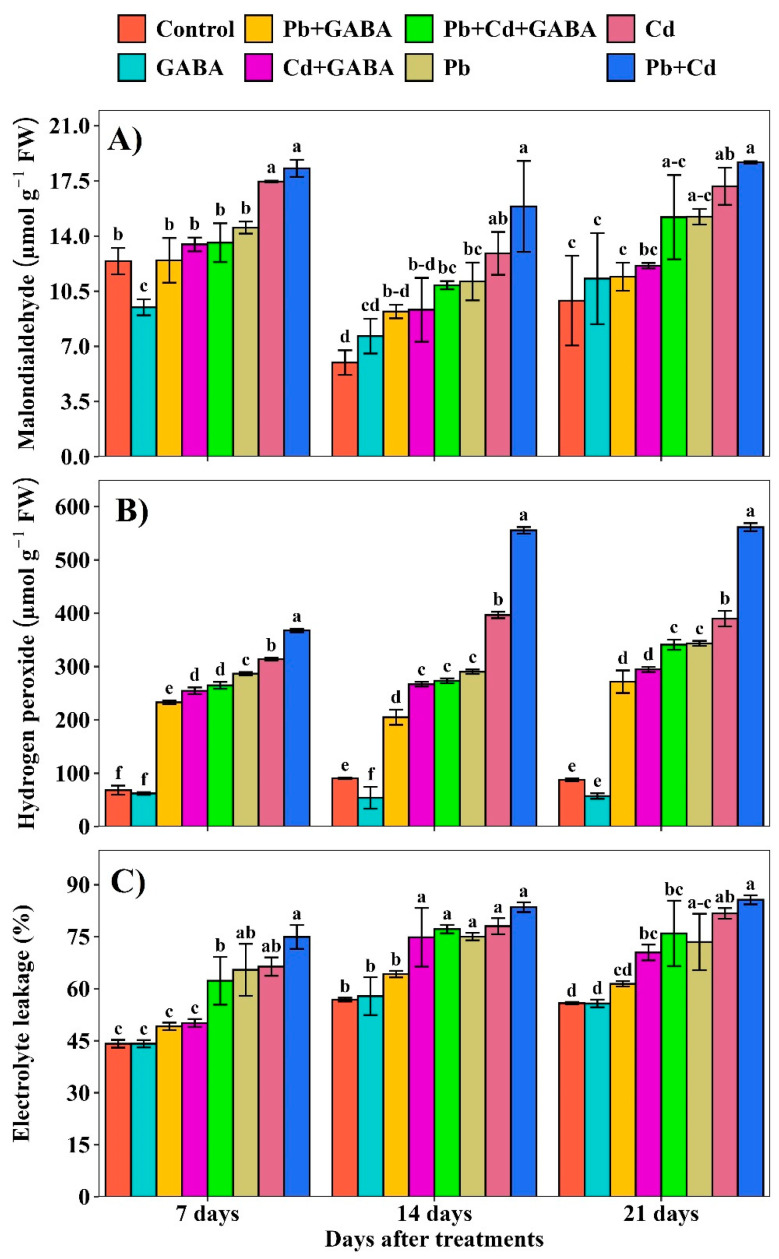
Effect of GABA application on (**A**) malondialdehyde (MDA) concentration, (**B**) H_2_O_2_ concentration, and (**C**) electrolyte leakage (EL) in rice at 7, 14, and 21 DAT under individual and combined Pb and Cd toxicity. Vertical bars (n = 4) with different lowercase letters differ significantly (*p* ˂ 0.05). Capped bars above means are standard error (SE). Control (no Pb, Cd, or GABA), GABA (1 mM), Pb (800 mg/kg of soil)+GABA, Cd (75 mg/kg of soil)+GABA, Pb+Cd+GABA, Pb, Cd, and Pb+Cd without GABA.

Compared with the control, the MDA concentration in the Pb, Cd, and Pb+Cd treatments without GABA were 17.10, 40.64, and 47.39% higher at 7 days after treatment (DAT); 86.12, 115.92, and 165.81% higher at 14 DAT; and 53.80, 73.28, and 88.63% higher at 21 DAT, whereas MDA concentration was lower in the same treatments with GABA application. The highest MDA concentration was detected in the combined Pb+Cd stress than in the individual Pb and Cd stress (Figure 2A). The EL increased under Pb, Cd, and combined Pb+Cd toxicity in both GABA-treated and untreated plants; however, the increase was more pronounced in plants that did not receive GABA. Compared with the control, the EL was increased by 48.32, 50.45, and 69.84% (without GABA) and 11.37, 13.61, and 41.19% (with GABA) at 7 DAT and 31.97, 37.25, and 46.88% (without GABA); 12.95, 31.62, and 35.77% (with GABA) at 14 DAT, and 31.60, 46.37, and 53.40% (without GABA); and 9.97, 26.17, and 36.06% (with GABA) at 21 DAT under Pb, Cd, and Pb+Cd toxicity, respectively (Figure 2B). Furthermore, H_2_O_2_ concentrations increased in response to Pb and Cd stress in both GABA-treated and untreated plants progressively from 7 to 21 DAT under Pb, Cd, and Pb+Cd stress than the control (Figure 2C).

### 2.2. Chlorophyll Contents and Carotenoids

**Figure 3:**
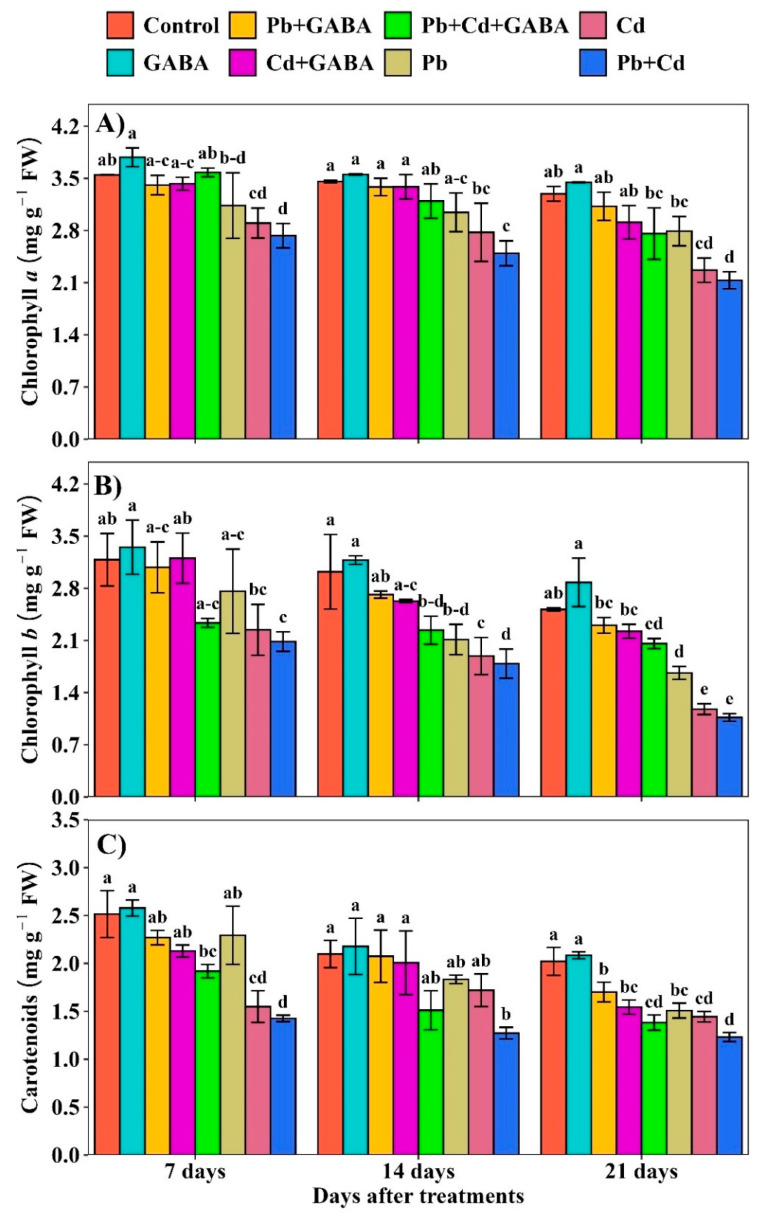
Effect of GABA application on (**A**) chlorophyll *a*, (**B**) chlorophyll *b*, and (**C**) carotenoid contents in aromatic rice at 7, 14, and 21 DAT under individual and combined Pb and Cd toxicity. Vertical bars (n = 4) with different lowercase letters differ significantly (*p* ˂ 0.05). Capped bars above means are standard error (SE). Control (no Pb, Cd, or GABA), GABA (1 mM), Pb (800 mg/kg of soil)+GABA, Cd (75 mg/kg of soil)+GABA, Pb+Cd+GABA, Pb, Cd, and Pb+Cd without GABA.

Compared with the control, the Chl *a*, Chl *b*, and carotenoids declined progressively from 7 to 21 DAT under Pb, Cd, and combined Pb+Cd stress. The highest reductions were noticed at 21 DAT in Pb, Cd, and Pb+Cd with and without GABA; nevertheless, the reductions were more pronounced in plants not treated with GABA. For example, at 21 DAT, the Chl *a*, Chl *b,* and carotenoids were reduced by 5.10%, 8.45%, and 15.84% under Pb, 11.60, 11.63, and 23.61% under Cd, and 16.22%, 18.21%, and 31.59% under Pb+Cd with GABA, respectively, compared to the control. In contrast, the reductions were more severe, i.e., 15.23%, 33.84%, and 25.40% under Pb, 31.13%, 53.16%, and 28.58% under Cd, and 35.27%, 57.61%, and 39.08% under Pb+Cd, respectively (Figure 3A–C).

### 2.3. Proline, Protein, and GABA Contents

**Figure 4:**
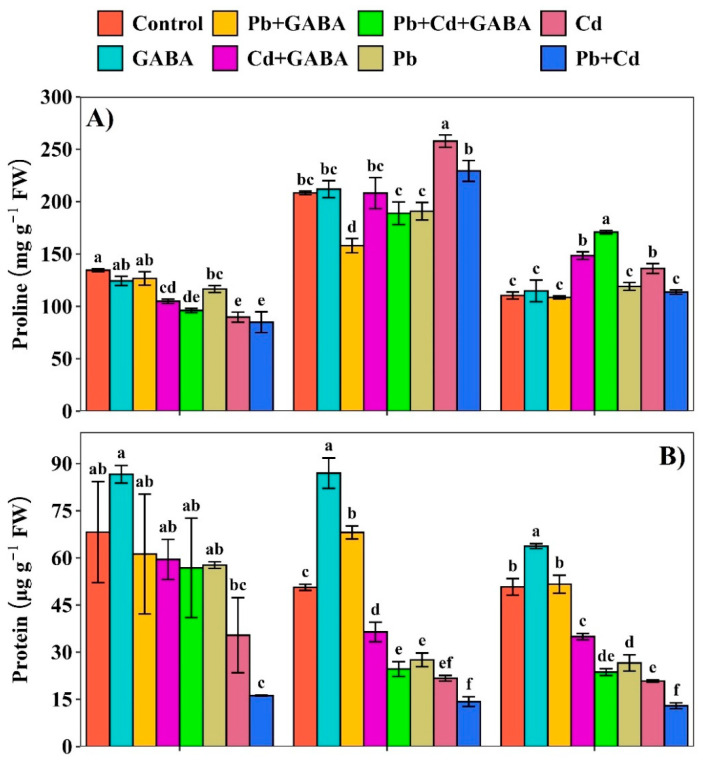

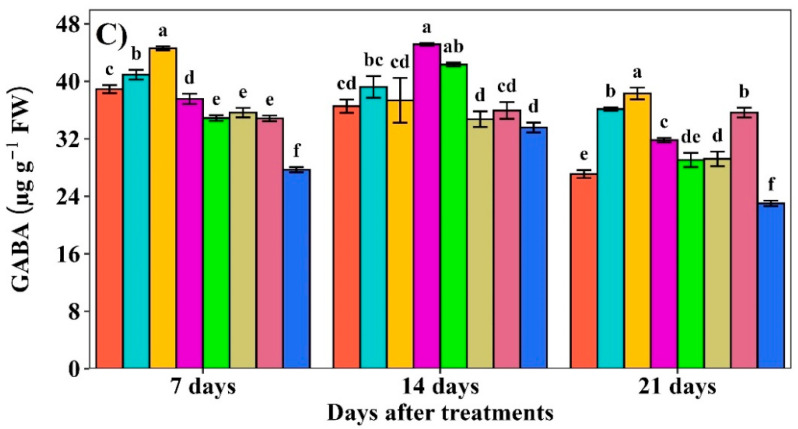
Effect of GABA application on (**A**) proline, (**B**) protein, and (**C**) GABA contents in aromatic rice at 7, 14, and 21 DAT under individual and combined Pb and Cd toxicity. Vertical bars (n = 4) with different lowercase letters differ significantly (*p* ˂ 0.05). Capped bars above means are standard error (SE). Control (no Pb, Cd, or GABA), GABA (1 mM), Pb (800 mg/kg of soil)+GABA, Cd (75 mg/kg of soil)+GABA, Pb+Cd+GABA, Pb, Cd, and Pb+Cd without GABA.

Compared with the control, the proline contents under Pb and Cd, either individual or combined, were substantially lower in both GABA-treated and non-treated plants. At 7 DAT, proline contents were significantly lower in the Cd and Pb+Cd treatments compared to the control, whereas the highest proline levels were observed in the GABA and Pb+GABA treatment. At 14 DAT, proline contents were the highest in the Cd treatment, followed by Pb+Cd, whereas at 21 DAT, proline levels peaked in the Pb+Cd+GABA treatment, followed by Cd+GABA and Cd treatments. Overall, the proline contents increased from 7 to 14 DAT and then decreased gradually until 21 DAT (Figure 4A). In contrast, Pb and Cd toxicity substantially reduced the protein contents, and the degree of damage was severe for non-GABA-treated plants under individual and combined Pb and Cd stress. Compared with the control, the protein contents in Cd+GABA, Pb+GABA, Pb, Cd, and Pb+Cd were reduced by 12.76%, 16.69%, 15.38%, 48.09%, and 76.31%, respectively, at 7 DAT; 28.07%, 51.36%, 45.59%, 57.15%, and 71.84%, respectively, at 14 DAT; and 31.15%, 53.42%, 47.67%, 58.98%, and 74.52%, respectively, at 21 DAT. In general, the protein contents remained higher at 7 and 14 DAT and then decreased at 21 DAT (Figure 4B). Exogenous GABA application modulated the endogenous GABA contents; nevertheless, the greatest reductions in GABA were recorded under Pb+Cd, i.e., 28.82, 8.12%, and 15.04% at 7, 14, and 21 DAT. At 7 DAT, the highest GABA content was detected in the GABA-alone (without Pb or Cd) treatment; however, the GABA contents were increased in all the GABA-treated plants but decreased significantly in non-GABA plants (Figure 4C).

### 2.4. Antioxidant Enzyme Activities

**Figure 5:**
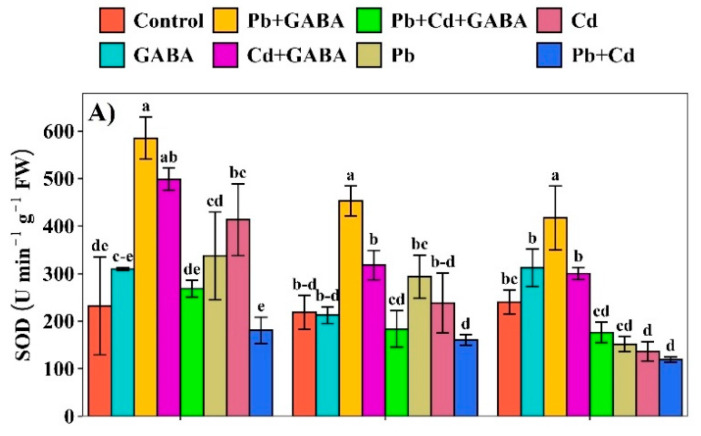

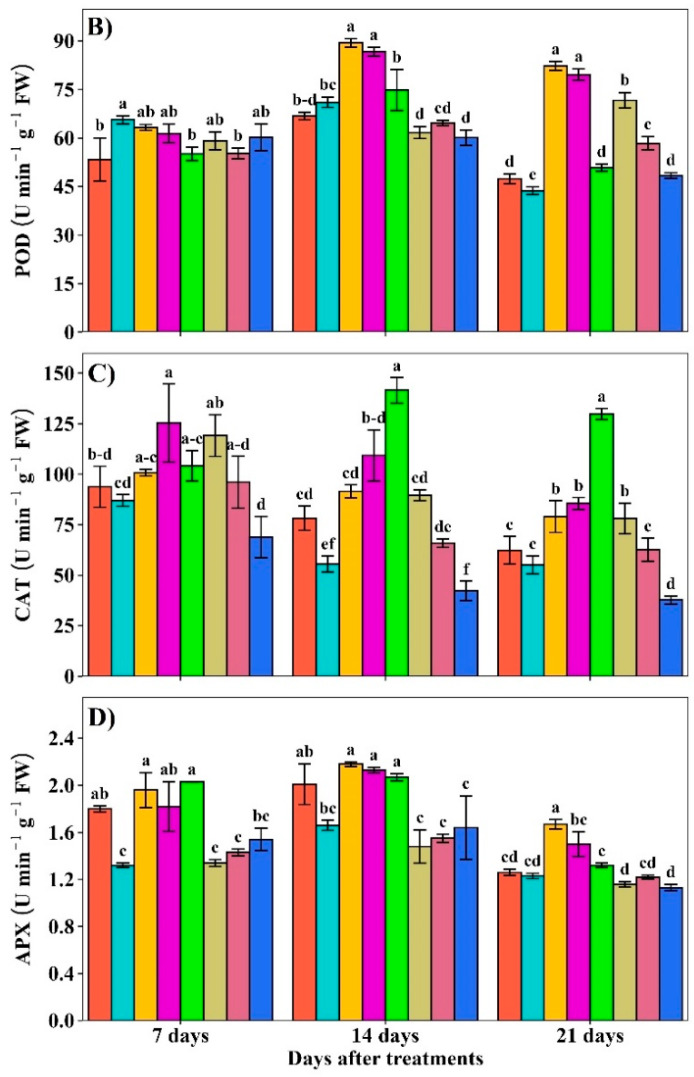
Effect of GABA application on (**A**) superoxide dismutase (SOD), (**B**) peroxidase (POD), (**C**) catalase (CAT), and (**D**) ascorbate peroxidase (APX) activities in rice at 7, 14, and 21 DAT under individual and combined Pb and Cd toxicity. Vertical bars (n = 4) with different lowercase letters differ significantly (*p* ˂ 0.05). Capped bars above means are standard error (SE). Control (no Pb, Cd, or GABA), GABA (1 mM), Pb (800 mg/kg of soil)+GABA, Cd (75 mg/kg of soil)+GABA, Pb+Cd+GABA, Pb, Cd, and Pb+Cd without GABA.

Compared to the control, the superoxide dismutase (SOD), peroxidase (POD), Ascorbate peroxidase (APX), and catalase (CAT) activities were generally higher in GABA-treated plants at 7, 14, and 21 DAT. The maximum inhibition of SOD activity was recorded in the Pb+Cd treatment, in which the SOD activity was 22.16%, 26.50%, and 50.46% lower at 7, 14, and 21 DAT than in the control (Figure 5A). The POD activity remained relatively higher than the control under all treatments, except for Pb, Cd, and Pb+Cd at 14 DAT, where it was reduced by 7.60%, 3.19%, and 10.02%, respectively. At 21 DAT, the POD activity was the lowest in the GABA-alone treatment compared to all other treatments (Figure 5B). Moreover, the highest CAT activity was found in GABA + Cd at 7 DAT and Pb+Cd+GABA at 14 and 21 DAT, i.e., 33.67%, 80.94, and 108.19% higher than the control, respectively, while the lowest CAT activity was recorded in Pb+Cd treatment at 7, 14, and 21 DAT (Figure 5C). GABA application modulated APX activity under both individual and combined Pb and Cd stress. In contrast, under non-GABA treatments, the APX activity was substantially reduced. Cd alone led to the highest inhibitions in APX activities, i.e., 25.82% and 26.45% at 7 and 14, and 9.79% at 21 DAT, respectively, as compared to the control (Figure 5D).

### 2.5. GSH Contents and GS and NR Activities

**Figure 6:**
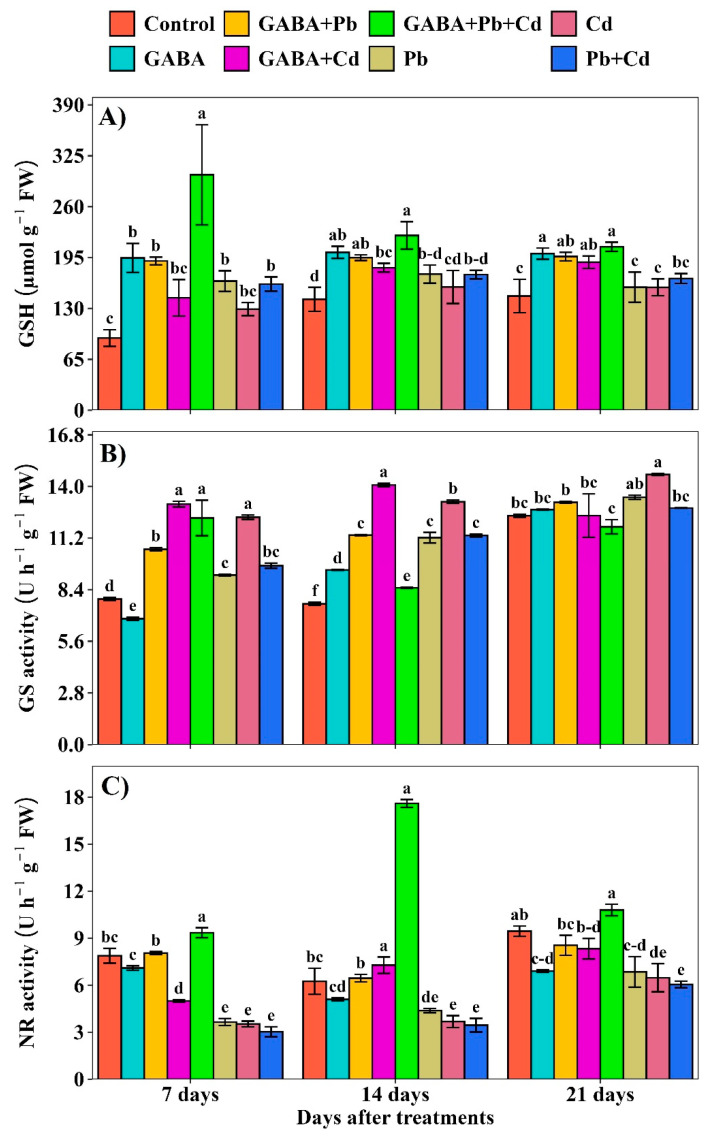
Effect of GABA on (**A**) reduced glutathione (GSH) concentration and (**B**) glutamine synthetase (GS) and (**C**) nitrate reductase (NR) activities in rice at 7, 14, and 21 DAT under individual and combined Pb and Cd toxicity. Vertical bars (n = 4) with different lowercase letters differ significantly (*p* ˂ 0.05). Capped bars above means are standard error (SE). Control (no Pb, Cd, or GABA), GABA (1 mM), Pb (800 mg/kg of soil)+GABA, Cd (75 mg/kg of soil)+GABA, Pb+Cd+GABA, Pb, Cd, and Pb+Cd without GABA.

The GSH contents were increased under individual and combined Pb and Cd treatments but the increment was obviously higher in GABA+Pb+Cd, whereas the lowest GSH contents were recorded in the control at 7, 14, and 21 DAT (Figure 6A). GABA application regulated GS and NR activities under Pb and Cd toxicity. The maximum GS activity was noted under the Cd+GABA treatment while the minimum was observed under the control and GABA-alone at 7 and 14 DAT, respectively. At 21 DAT, maximum GS activity was observed in Cd alone followed by Pb alone, while the lowest was found under Pb+Cd+GABA treatment (Figure 6B). Furthermore, NR activity also exhibited a differential response to Pb and Cd stress in response to GABA application. The NR activity was consistently lower in plants without GABA under Pb, Cd, and Pb+Cd treatments at 7, 14, and 21 DAT. The highest NR activity was observed in the Pb+Cd+GABA treatment, which was 18.73%, 181.60%, and 14.29% higher than the control, and 209.09%, 410.15%, and 78.15% higher than the Pb+Cd treatment at 7, 14, and 21 DAT, respectively (Figure 6C).

### 2.6. Net Photosynthesis and Gas Exchange Characteristics

**Figure 7:**
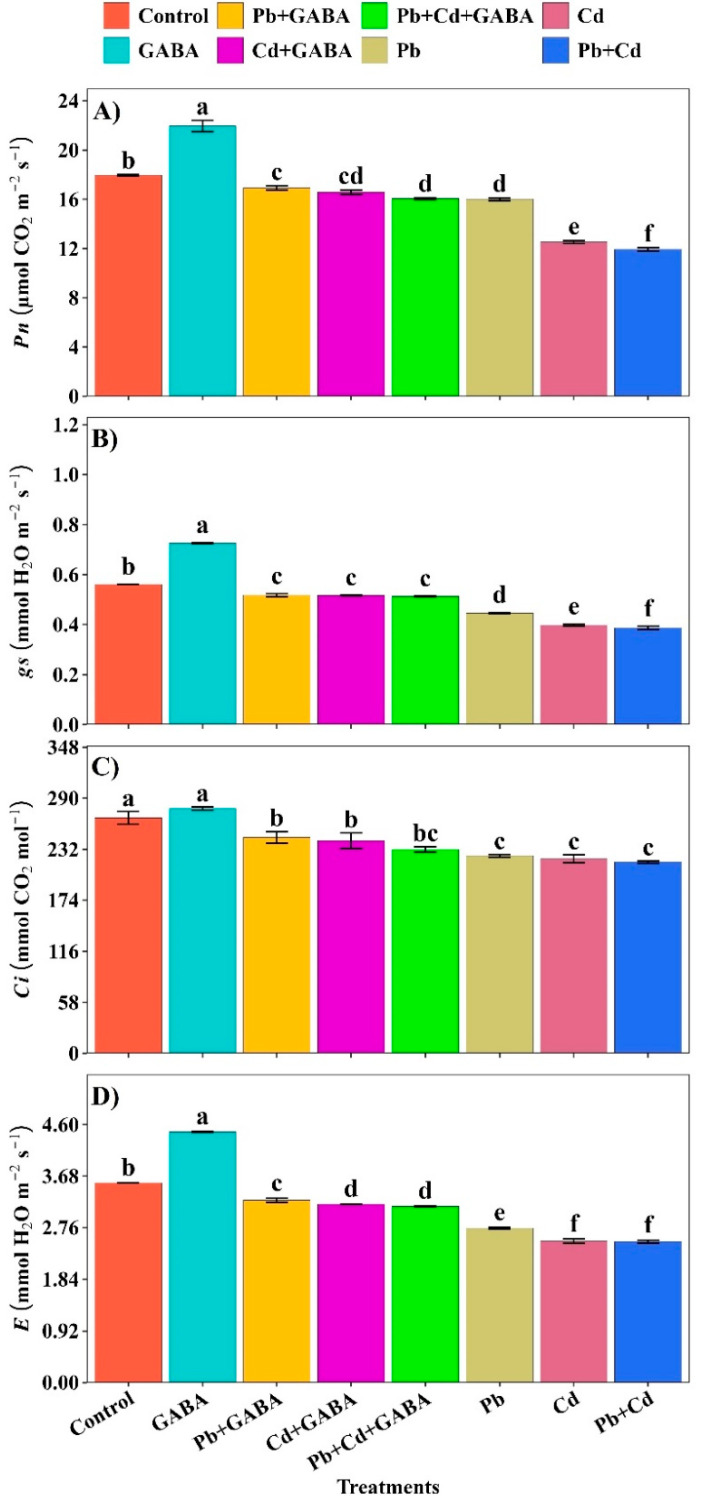
Effect of GABA application on (**A**) net photosynthesis (*Pn*), (**B**) stomatal conductance (*gs*), (**C**) intercellular CO_2_ (*Ci*), and (**D**) transpiration rate (*E*) in rice under individual and combined Pb and Cd toxicity. Vertical bars (n = 4) with different lowercase letters differ significantly (*p* ˂ 0.05). Capped bars above means are standard error (SE). Control (no Pb, Cd, or GABA), GABA (1 mM), Pb (800 mg/kg of soil)+GABA, Cd (75 mg/kg of soil)+GABA, Pb+Cd+GABA, Pb, Cd, and Pb+Cd without GABA.

Compared with the control, the highest net photosynthesis (*Pn*), stomatal conductance (*gs*), intercellular CO_2_ (*Ci*) and transpiration rate (*E*) were recorded in plants not exposed to Pb, Cd, or Pb+Cd. The decreasing trends of these physiological traits under the treatments were as follows: GABA > Control > Pb+GABA > Cd+GABA > Pb+Cd+GABA > Pb > Cd > Pb+Cd. Hence, individual or combined Pb and Cd toxicity severely impaired the photosynthetic performance of aromatic rice; however, exogenous GABA application partially mitigated these adverse effects (Figure 7A–D).

### 2.7. Grain Yield

**Figure 8:**
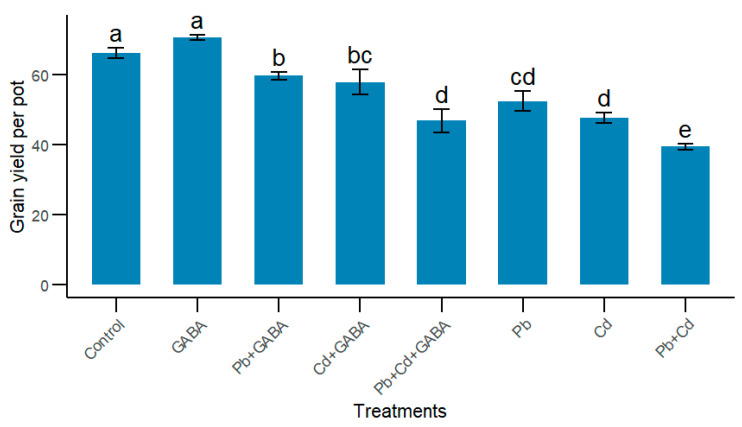
Effect of GABA application on grain yield of rice under individual and combined Pb and Cd toxicity. Vertical bars (n = 4) with different lowercase letters differ significantly (*p* ˂ 0.05). Capped bars above means are standard error (SE). Control (no Pb, Cd, or GABA), GABA (1 mM), Pb (800 mg/kg of soil)+GABA, Cd (75 mg/kg of soil)+GABA, Pb+Cd+GABA, Pb, Cd, and Pb+Cd without GABA.

Individual and concurrent Pb and Cd stress significantly reduced the grain yield, while exogenous GABA application improved the grain yield under Pb and Cd toxicity conditions. For instance, compared to the Pb+Cd treatment, the grain yield was 34.03%, 31.94%, 15.88%, 24.86%, and 17.32% higher in the Pb+GABA, Cd+GABA, Pb+Cd+GABA, Pb, and Cd treatments, respectively. The maximum grain yield was recorded in the GABA treatment, which was statistically similar (*p* > 0.05) to the control (Figure 8).

### 2.8. Mineral Nutrients

**Figure 9:**
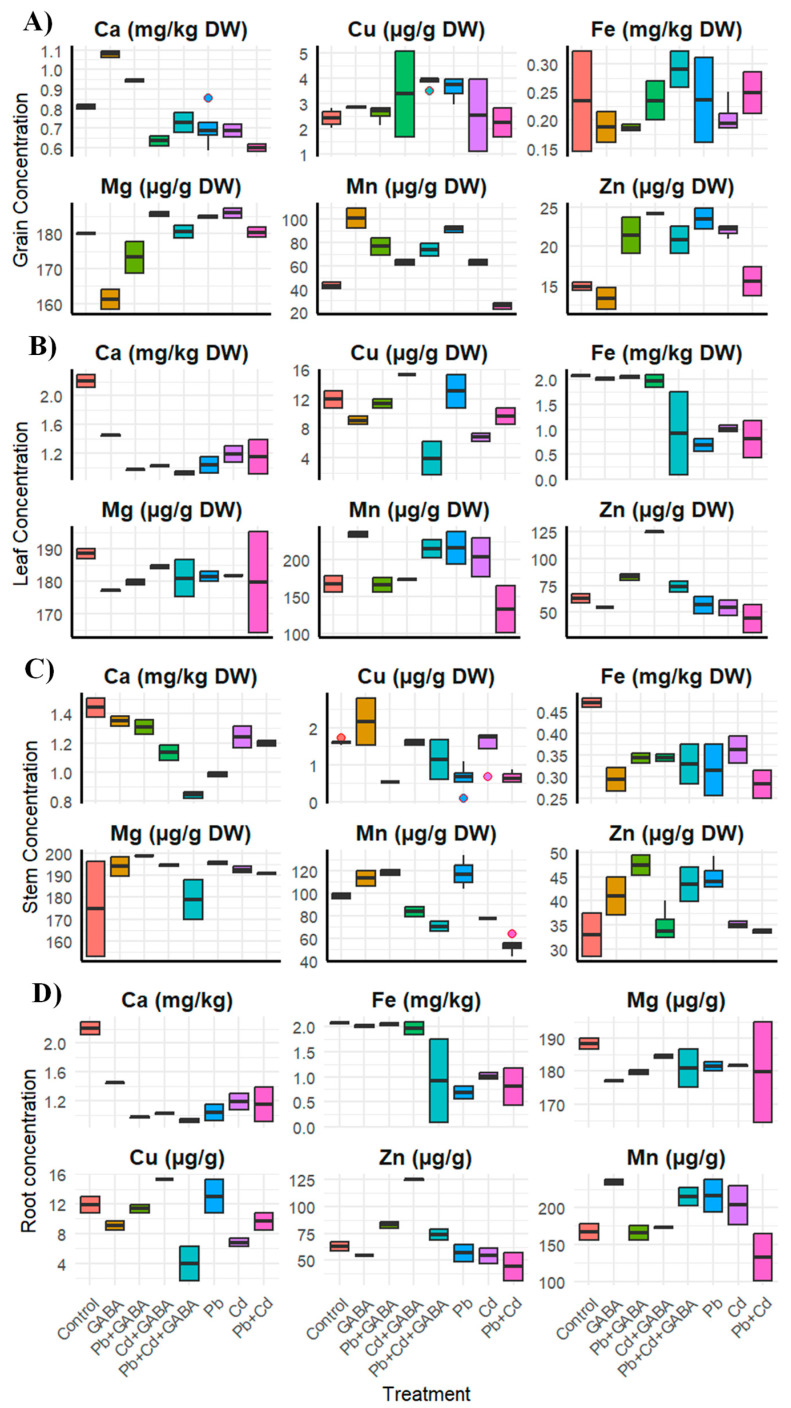
Effects of GABA application on contents of mineral nutrients, i.e., Ca, Mg, Cu, Mn, Fe, and Zn in (**A**) grains, (**B**) leaves, (**C**) stems, and (**D**) roots of aromatic rice under individual and combined Pb and Cd stress. Dots outside box are ‘outliers’ while bars inserted in boxes are ‘whiskers’. Control (no Pb, Cd, or GABA), GABA (1 mM), Pb (800 mg/kg of soil)+GABA, Cd (75 mg/kg of soil)+GABA, Pb+Cd+GABA, Pb, Cd, and Pb+Cd without GABA.

Exogenous GABA application modulated the mineral nutrients in different plant parts under Pb and Cd stress. Pb and Cd altered macro- and micronutrient contents across roots, stems, leaves, and grains. Specifically, in grains, Ca and Fe levels substantially reduced under all stress treatments, especially in combined Pb+Cd, while Cu and Mn showed variable responses across all treatments. Mg increased under Cd and Pb+Cd whilst Zn sharply declined under Pb+Cd (Figure 9A). In leaves, Ca and Fe were significantly reduced by Pb and Cd, with the lowest Fe observed under Cd treatment. Cu and Mn contents decreased, especially under Pb+Cd, whereas Mg contents were marginally changed across all treatments. On the other hand, Zn contents were substantially declined under Pb and Cd exposures; nevertheless, GABA treatment showed substantial improvements in Ca, Cu, Fe, and Mn contents in leaves (Figure 9B). In stems, under Pb+Cd, Ca, Cu, and Mn were notably reduced, whereas GABA restored Cu and Mn under combined stress. Fe showed slight reductions, while Zn increased under Pb but declined under Cd and Pb+Cd. It was further noticed that Mg contents remained statistically similar across all treatments (Figure 9C). In addition, Ca and Fe declined sharply with heavy metal stress, particularly Cd and Pb+Cd in roots. Interestingly, Cu and Mn levels increased, especially under Pb, whilst Zn declined substantially under individual and combined Pb and Cd toxicity. It was further noticed the Mg contents were quite similar across all treatments. Moreover, GABA application improved Ca, Fe, and Zn uptake under metal stress, especially under Pb+Cd (Figure 9D). Overall, Pb and Cd stress inhibited nutrient accumulation in aboveground plant parts of aromatic rice, while GABA ameliorated most nutrient deficiencies, particularly in Ca, Fe, and Zn.

### 2.9. Pb and Cd Accumulation in Different Plant Parts and Translocation Factor

The Pb and Cd concentrations were readily increased in the roots, leaves, stems, and grains in the Pb/Cd treatment group; however, GABA application reduced both the Pb and Cd concentrations in all the plant parts, including the roots. The concentration of both Pb and Cd were the highest in the roots, followed by the stems, leaves, and grains. Compared to the Pb treatment, the Pb concentration in the combined Pb+Cd treatment was lower in all plant parts; however, these reductions were even more prominent in the applied GABA treatments. The Pb concentration was reduced by 32.98%, 32.09% and 24.76% in roots, 13.63%, 33.87% and 3.67% in stems, 35.29%, 40.03% and 26.93% in leaves, and 47.12%, 48.08%, 8.72% in grains under Pb+GABA, Pb+Cd+GABA and Pb+Cd than individual Pb treatment. Likewise, compared to the Cd treatment, the Cd concentration was also reduced by 11.14%, 13.59% and 8.71% in roots, 42.95%, 51.44% and 15.92% in stems, 22.88%, 30.51% and 50.00% in leaves, and 16.78%, 33.41% and 27.12% in grains under Cd+GABA, Pb+Cd+GABA and Pb+Cd treatments, respectively. Overall, the Pb and Cd concentrations remained lower in the combined Pb+Cd treatment than in the individual Pb and Cd treatments; however, exogenous GABA application further reduced the Pb and Cd concentrations in the different plant parts of rice (Table 1). In addition, mean values of translocation factor (TF) across all treatments were the highest from stems-to-leaves (0.6849 and 0.6887) than roots-to-stems (0.1555 and 0.4838) and leaves-to-grains (0.0470 and 0.0294) for both metals, i.e., Pb and Cd, respectively. Moreover, the mean translocation factor (TF) values for roots-to-stems and stems-to-leaves were generally higher for Cd than Pb, whereas Pb showed higher TF values than Cd for leaves-to-grains (Table 2).

### 2.10. Correlation Analysis

**Figure 10:**
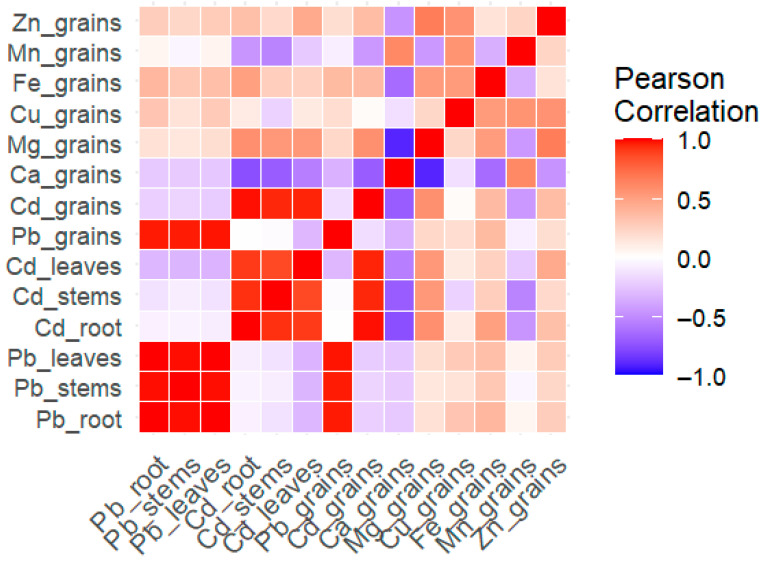
Pearson correlation of Pb and Cd concentrations in roots, stems, and leaves of Pb, Cd, Zn, Fe, Mn, Cu, Mg, and Ca concentrations in rice grains. Intensity of color indicates strength of correlation. Pb_root, Pb_stems, Pb_leaves, Cd_root, Cd_stems, Cd_leaves, Ca_grains, Mg_grains, Cu_grains, Fe_grains, Mn_grains, and Zn_grains are Pb, Cd, Ca, Mg, Cu, Fe, Mn, and Zn contents in respective plant parts.

Pearson correlation heatmap revealed strong positive correlations among Pb and Cd contents in different tissues, i.e., roots, stems, leaves, and grains. Interestingly, Pb and Cd contents in roots, stems, and leaves showed strong positive associations with each other, while their correlations with essential elements were found generally weak or negative. Notably, Ca in grains exhibited a strong negative correlation with Cd in grains (r < –0.7), indicating a potential antagonistic interaction. Additionally, Cu in grains displayed moderate positive correlations with Fe and Zn contents in grains, implying synergistic relationships among both micronutrients in grains. The overall correlation pattern suggested that the translocation and accumulation of Pb and Cd are strongly coordinated within plant organs, while the presence of these heavy metals may disrupt the uptake or distribution of essential plant nutrients (Figure 10).

## 3. Discussion

Increasing soil contamination with heavy metals poses a serious challenge to quality food production. Once the permissible limits are exceeded, Pb and Cd can exert severe toxic effects on both plants and human health. This study investigated the role of exogenous GABA application in mitigating the negative effects of Pb and Cd stress. Notably, both Pb and Cd, alone or in combination, increased oxidative damage in rice, whereas exogenous GABA application reduced lipid peroxidation (decreased malondialdehyde (MDA) concentration), electrolyte leakage, and H_2_O_2_ levels (Figure 2A–C). A higher MDA indicates the extent of lipid peroxidation because Pb/Cd induces excessive reactive oxygen species (ROS), which can be damaged by oxidizing lipids. MDA is a mutagenic carbonyl compound that is a sign of stress in plants and is produced as a result of the oxidation of polyunsaturated fats, whereas EL indicates a decrease in membrane integrity, which was enhanced substantially under Pb/Cd toxicity conditions. Moreover, Pb/Cd stress in rice also promoted H_2_O_2_, a stress signaling molecule at low concentrations, but its overproduction caused significant damage due to its oxidizing properties [20]. Exogenous GABA application reduced ROS-induced oxidative stress through a scavenging mechanism. Previously, reduced lipid peroxidation due to GABA application under Al^3+^ and H^+^ stress was also observed in barley [21] and maize [22]. Similarly, GABA priming in black pepper plants led to significant reductions in MDA biosynthesis but increased proline and soluble sugar contents under polyethylene glycol (PEG)-induced stress [23].

The alleviative effect of GABA on Pb- and Cd-induced oxidative stress in rice could be attributed to its role in enhancing the antioxidant defense system, as shown in Figure 5. Exogenous GABA generally upregulates the activities of key ROS scavenging enzymes, thus reducing oxidative damage. Additionally, GABA may help maintain redox homeostasis by modulating non-enzymatic antioxidants and limiting Pb and Cd uptake and translocation. Its involvement in the GABA shunt and stress-responsive signaling further supports its protective role under metal stress conditions. These findings are consistent with previous reports demonstrating GABA-mediated oxidative stress mitigation in plants under abiotic stress [24].

Both Pb and Cd reduced photosynthetic pigments, photosynthesis, and gas exchange, whereas exogenously applied GABA enriched the chlorophyll content and net photosynthesis under individual and combined Pb and Cd toxicity (Figure 3A–C and Figure 7A–D). Heavy metal toxicity generally inhibits chlorophyll biosynthesis and carotenoids and causes ultrastructural changes due to the overproduction of ROS, thus leading to reduced photosynthesis [25,26]; however, GABA probably inhibited chlorophyll degradation and improved net photosynthesis under Pb/Cd stress. Pb/Cd toxicity significantly affected the proline, protein, and endogenous GABA contents in rice (Figure 4A–C). An increase in proline contents in GABA-treated plants is an adaptive response of rice plants under Pb/Cd stress and has an osmoregulatory and ROS scavenging role, while an increase in endogenous GABA under GABA treatment might be due to direct absorption from foliar application as well as an increase in the rate of biosynthesis through the GABA shunt. GABA protected the proteins from denaturation, possibly due to its interference with Pb- or Cd-induced ROS production while improving antioxidant activities (POD, SOD, APX, CAT, and GSH) under individual and combined Pb and Cd stress conditions (Figure 5). Plants normally activate their ROS scavenging mechanism under stress conditions, for instance, antioxidant activities increase with increasing metal stress [27], while others have reported that severe toxic conditions lead to significant reductions in antioxidant activities [28]. In the present study, antioxidant enzyme activities were lower under individual or combined Pb and Cd stress conditions (without GABA), while exogenous GABA application modulated the activities of these enzymes, thus helping to eliminate ROS. O_2_^−^ is detoxified to O_2_, followed by H_2_O_2_, which is further reduced to H_2_O by the interlinked mechanism of SOD, POD, and CAT to counteract the production and accumulation of ROS. A decrease in antioxidant activity under Pb/Cd stress without GABA reduced the defense efficiency of the plants with respect to the production rate of ROS. GABA application also modulates the expression of γ-aminobutyric acid transaminase *(OsGABA-T*) and succinic semialdehyde dehydrogenase (*OsSSADH*) genes, indicating its role in enhancing copper (Cu) stress resilience in rice [29].

In addition, GABA acts not only as a metabolite and signaling molecule but also interacts synergistically with key phytohormones to enhance rice tolerance under Pb and Cd stress. The interplay between GABA and various plant hormones including abscisic acid, cytokinins, auxins, gibberellins, and ethylene plays a crucial role in regulating the expression of stress-responsive genes, thus helping plants cope with multiple abiotic stresses [30]. Furthermore, GABA application substantially modulated the multiple phytohormones and related compounds, including benzoic acid, salicylic acid, cinnamic acid, indole acetic acid, trans-jasmonic acid, abscisic acid, and indole propionic acid in *Citrus sinensis* [31]. This complex hormonal interplay governed by GABA suggests a more robust antioxidant response, metal sequestration, and cellular homeostasis and underscores the multifaceted role of GABA as a modulator of phytohormonal networks in mitigating heavy metal stress in rice.

In addition, Pb/Cd interfered with normal C-N metabolism by disturbing GS and NR activities in rice; nevertheless, exogenous GABA application helped to maintain GS and NR activities. Moreover, the effects of Pb/Cd toxicity on NR activity were more severe than those on GS activity; however, exogenous GABA application improved GS and NR activity (Figure 6B,C). Both GS and NR are key enzymes involved in nitrogen metabolism and the conversion of complex and organic forms of nitrogen to simple inorganic compounds [32]. Reduced NR activity in Pb/Cd plants possibly decreased the reduction rate of nitrate to nitrite, thus inhibiting plant growth, while reduced GS activity led to disturbed C metabolism. Hence, GABA helps plants maintain a balance between GS and NR under Pb/Cd stress. NR and GS activities decreased in response to heavy metal toxicity [33]. Furthermore, the roles of GABA in maintaining the C:N balance were reported by Fromm [34]. Pb and Cd, either individually or in combination, reduced grain yield (Figure 8). The reduced grain yields in rice might be due to impaired physio-biochemical mechanisms, reduced photosynthesis, and increased ROS activity, while GABA induces the modulation of antioxidant defense and maintains GS and NR activity, and osmoregulation leads to yield improvements even under Pb/Cd toxicity. Exogenous GABA application enhances the physio-biochemical and yield attributes of rice under normal and heavy metal toxicity conditions [35].

Exogenous GABA application also regulated the Cu, Fe, Zn, and Mn levels in the roots, stems, leaves, and grains under Pb and Cd toxicity, whereas the mineral nutrient levels were lower in the Pb and Cd treatments (Figure 9A–D), which might be due to antagonistic effects. Regarding mineral nutrients, both root Pb and Cd showed positive associations with stem, leaf, and grain Pb and Cd concentrations, respectively, which may compete with essential micronutrients, and could have synergistic and/or antagonistic effects on other essential mineral nutrients (Figure 10). Plants often take up essential and nonessential micronutrients from the soil via selective uptake and/or following a concentration gradient; however, transporters also play crucial roles in the uptake and translocation of metals in ionic form, e.g., IRT and ZRT (Fe/Zn regulated transporters, respectively) and mediate the transfer of several metals, including Fe, Zn, Mn, and Cd [36,37]. Liu et al. [38] reported the positive interactions of Cd with Fe, Zn, and Cu in rice and reported synergistic effects on root-to-shoot uptake and translocation in rice.

In addition, roots are the first organs that contact metals such as Pb and Cd and translocate these metals to upper plant parts. It was found that, under the Pb and Cd treatments, the contents of the respective metals were higher when the plants were treated with GABA (Table 1), whereas the values of TF were higher for Cd in roots-to-stems and stems-to-leaves but were higher for Pb in leaves-to-grains (Table 2). Hence, there is a possibility of competition between Pb and Cd metals that hinders the entry and/or uptake of one metal over the other. Cd is comparatively more readily taken up by plant roots than Pb is due to Ca^2+^, Zn^2+^, and Fe^2+^ ions and transporters [39,40]; on the other hand, Cd is taken up by plants via the apoplastic pathway and/or permeable Ca^2+^ channels in plant roots [41]. Moreover, blockage of Pb within the endodermis in roots, clinging of Pb into vascular bundles, bonding of Pb ions by negative charges on the root cell wall, and/or adsorption on the root surface are key factors that cause its uptake and movement within plants to be slower than those of Cd [42,43]. Similarly, Khan et al. [44] reported that GABA reduced Ni accumulation by enhancing the expression of metal transporter proteins *OsMTP1* and *OsMTP8*, which facilitated Ni sequestration into vacuoles and helped restore essential mineral levels including Ca^2+^ and Mg^2+^.

While the physiological benefits of exogenous GABA application in mitigating Pb and Cd stress in rice are well documented, its economic feasibility and potential for large-scale field adoption remain critical considerations. GABA is relatively affordable and available commercially and can potentially be used as plant growth supplements as a promising option, compared to synthetic chelators and/or advanced remediation technologies. Moreover, its compatibility with foliar spray and fertigation methods allows for easy integration into existing farming practices without the need for significant infrastructure changes. Field trials have shown that low to moderate doses of GABA can improve the yield and quality of aromatic rice [45]. However, adoption at the farm level would require dose optimization, application timing, and regulatory compliance to ensure consistency and environmental safety. Therefore, integrating GABA-based strategies with conventional agronomic practices could be a promising and economically viable approach for improving rice yield under multiple abiotic stresses.

## 4. Materials and Methods

### 4.1. Experimental Description

A pot experiment was performed in a greenhouse from April to July 2016 (early season rice) at the Experimental Site, South China Agricultural University (SCAU), Guangzhou, China (altitude 11 m and 23°09′ North, 113°22′ East). The germinated seeds of the rice cultivar ‘Guixiangzhan’ (regionally famous aromatic rice cultivar) were sown in soil-filled seedling trays in early March and covered for growing rice seedlings. Twenty-five days before transplanting rice seedlings, the air-dried soil was filled in to 25 cm × 32 cm pots (height × diameter) at a rate of 10 kg per pot. Pb and Cd, at 800 mg and 75 mg per kg of soil in the form of Pb(NO_3_)_2_ and CdCl_2_ solutions, respectively, were added and thoroughly mixed into the soil [38]. The experimental treatments are described in Table 3. Pb and Cd above this level were proven to be harmful to aromatic rice (preliminary trials). Rice plants (5 hills and 2 seedlings per hill) were transplanted into pots, and NPK was applied at 2.25, 3.30, and 1.40 g per pot in the form of urea (46% N), superphosphate (12% P_2_O_5_), and potassium chloride (60% K_2_O), respectively. The pots were irrigated regularly by maintaining a 2–3 cm water layer. Gamma-aminobutyric acid (GABA) (1 mM) was applied thoroughly at the active tillering stage (21 days after transplanting) two times at three-day intervals based on the reports of Nayyar et al. [46], who reported that 1 mM GABA was the most effective treatment for rice under stress conditions. The experimental soil was sandy loam comprising 1.02 g of total nitrogen and 79.74 mg of available nitrogen, 0.93 g of total phosphorus and 9.73 mg of available phosphorus, 17.60 g of total potassium and 120.47 mg of available potassium, and 20.78 g of organic matter kg^−1^ of soil with a pH of 5.58. The initial soil Pb and Cd contents were 48.81 and 3.96 mg kg^−1^ soil, respectively.

### 4.2. Sampling and Measurements

Plant leaves were collected three times after GABA application (at 7, 14, and 21 days after treatment (DAT)) for plant physio-biochemical assays, whereas the plants were sampled at the maturity stage to determine the Pb, Cd, Ca, Mg, Cu, Zn, Fe, and Mn contents.

#### 4.2.1. Determination of Oxidative Stress Indicators

Malondialdehyde (MDA) concentration in leaves was estimated by following the protocols of Hodges et al. [47]. Briefly, fresh leaf tissue (0.2 g) was homogenized with a pestle and mortar in 2 mL of 0.5% thiobarbituric acid (TBA) prepared in 10% trichloroacetic acid (TCA), followed by heating at 100 °C for 30 min. After rapid cooling in an ice bath, the samples were centrifuged at 4000 g for 15 min. Absorbance was recorded at 532, 600, and 450 nm. Malondialdehyde (MDA) content was calculated using the formula MDA = [6.45 × (OD_532_ − OD600)] − (0.56 × OD_450_) and expressed as μmol g^−1^ fresh weight.

Hydrogen peroxide (H_2_O_2_) was determined according to Velikova et al. [48]. Fresh leaf samples (0.2 g) were homogenized in 1 mL of 0.1% trichloroacetic acid (TCA) and centrifuged by using a refrigerated centrifuge machine (Thermo Fisher Scientific, Waltham, MA, USA) at 12,000 g for 15 min. The reaction mixture comprised 0.5 mL of potassium phosphate buffer (pH 7.0), 1 mL of 1 M potassium iodide (KI), and 0.5 mL of the supernatant. Absorbance was measured at 390 nm in triplicate, and hydrogen peroxide (H_2_O_2_) content was expressed as µmol g^−1^ fresh weight.

Electrolyte leakage (EL) was estimated according to Valentovic et al. [49]. Briefly, fresh leaf disks (0.3 g) were put into 10 mL of deionized water at 25 °C for 6 h to record the initial electrical conductivity (EC1). The samples were then heated at 90 °C for 2 h, cooled to 25 °C, and final conductivity (EC2) was measured. The EL was calculated by using the following formula: EL (%) = (EC1/EC2) × 100.

#### 4.2.2. Estimation of Photosynthetic Pigments and GABA, Proline, and Protein Contents

Photosynthetic pigments, i.e., chlorophyll a (Chl *a*), chlorophyll b (Chl *b*), and carotenoids, were estimated according to Arnon [50]. Briefly, fresh leaf samples (0.2 g) were extracted with 10 mL of 95% ethanol and kept in the dark at room temperature overnight. The extracts were then filtered and absorbance was read at 665, 649, and 470 nm. The GABA contents were assessed in leaves by following the protocols of Yao et al. [51]. GABA was extracted from fresh leaves (0.2 g) using 60% ethanol, shaken for 4 h (HZS-H, Changzhou, China), and centrifuged at 8000× *g* for 5 min and 1 mL of the supernatant was mixed with 0.6 mL of 0.2 M sodium tetraborate, 2 mL of 5% phenol, and 1 mL of 7% sodium hypochlorite. The mixture was heated at 100 °C then cooled and the absorbance was measured at 645 nm. The GABA content was quantified using a standard curve and expressed as μg g^−1^ fresh weight. Leaf protein were assessed using Coomassie Brilliant Blue G-250 dye according to Bradford [52]. Fresh leaves (0.2 g) were homogenized in 50 mM sodium phosphate buffer (pH 7.0) containing 2% PVP-40 and 1 mM EDTA-Na_2_. The extract was centrifuged at 10,000× *g* for 15 min at 4 °C. The absorbance was recorded at 595 nm and protein content was expressed as µg g^−1^ fresh weight. The leaf proline contents were quantified by using ninhydrin according to Bates et al. [53] by using a spectrophotometer (UV-vis 2550, Shimadzu, Kyoto, Japan).

#### 4.2.3. Estimation of Reduced Glutathione (GSH) and Enzymatic Antioxidants

After 0.3 g of fresh leaves was homogenized in 6 mL of 50 mM sodium phosphate buffer (pH 7.8), the solution was centrifuged using a refrigerated centrifuge machine (Thermo Fisher Scientific, Waltham, MA, USA) at 8000× *g* for 20 min at 4 °C. The antioxidant enzyme activities and reduced glutathione (GSH) concentrations were measured in the supernatant.

Reduced glutathione (GSH) and ascorbate peroxidase (APX) were measured using premade kits acquired from Nanjing Jiancheng Bioengineering Institute in China (www.njjcbio.com). The superoxide dismutase (SOD), according to Zhang et al. [54], was based on the inhibition of nitro blue tetrazolium (NBT) photoreduction. Briefly, the reaction mixture included 1.75 mL of sodium phosphate buffer (pH 7.8) and 0.3 mL each of 130 mM methionine, 750 μM NBT, 100 mM EDTA-Na_2_, 20 μM riboflavin, and 0.05 mL enzyme extract. The absorbance was recorded at 560 nm. The quantity of enzyme needed to prevent NBT photochemical degradation to 50% as an activity unit (U) was known as SOD activity per unit. Peroxidase (POD) activity was estimated using the guaiacol method with slight modifications [55]. The reaction mixture contained 1 mL of sodium phosphate buffer (pH 7.8), 0.95 mL of 0.2% guaiacol, 1 mL of 0.3% H_2_O_2_, and 0.05 mL of enzyme extract. The absorbance was recorded at 470 nm. One unit of POD activity was the amount of enzyme catalyzing the decomposition of 1 mg of substrate at 470 nm. The catalase (CAT) activities were assessed according to Aebi [56]. The reaction mixture contained 1.95 mL of sodium phosphate buffer (pH 7.8), 1 mL of 0.1 M H_2_O_2_, and 0.05 mL of enzyme extract and the absorbance was measured at 240 nm. One unit of enzyme activity (U) was the amount decomposing 1 M H_2_O_2_ per minute per gram of fresh leaf tissue at A_240_. The absorbance was read by using a spectrophotometer (UV-vis 2550, Shimadzu, Kyoto, Japan).

#### 4.2.4. Determination of Nitrogen-Metabolizing Enzymes

The activity of glutamine synthetase (GS) was measured in accordance with previous methods of Wilson and Walker [57], whereas the nitrate reductase (NR) activity was measured according to Yu and Zhang [58] by using a spectrophotometer (UV-vis 2550, Shimadzu, Kyoto, Japan) (details in the Supplementary Materials).

### 4.3. Measurements of Photosynthesis and Gas Exchange Parameters

Net photosynthesis (*A*) and gas exchange properties, such as stomatal conductance (*gs*), intercellular CO_2_, and transpiration rate (*E*), were measured in flag leaves from 09:00 to 11:30 a.m. from each treatment by using a portable photosynthetic system (LI-6400, LI-COR, Lincoln, NE, USA) with the following adjustments: photosynthetically active radiation at leaf surface was up to 1200 μmol m^−2^ s^−1^, molar flow of air per unit leaf area was about 500 μmol s^−1^, ambient CO_2_ concentration was almost 400 μmol mol^−1^, and air temperature was about 28–30 °C with 70–85% relative humidity.

### 4.4. Determination of Pb and Cd Contents, Elemental Analysis, and Translocation Factor

At maturity, plants were carefully removed from the pots, thoroughly washed, and separated into leaves, roots, stems, and grains. The plant parts were then oven-dried at 80 °C. The dried samples were ground into fine powder, and 0.2 g of each was digested in a digestion block (SH220F Kjeldahl Digestion System, Shanghai Precision & Scientific Instrument Co., Ltd., Shanghai, China) using HNO_3_:HClO_4_ (4:1 *V/V*). The digested solutions were then filtered and diluted to a volume of 25 mL using deionized water. The contents of Pb and Cd as well as the elemental contents of Ca, Mg, Cu, Zn, Fe, and Mn in the corresponding samples were measured using an atomic absorption spectrophotometer (AA6300C, Shimadzu, Kyoto, Japan). The translocation factor (TF) was determined as the ratios of Pb and Cd content in roots, stems, leaves, and grains as root/stems, leaves/stems, and grains/leaves.

### 4.5. Determination of Grain Yield

At maturity, the grains from all the remaining pots were manually collected, threshed, and sun-dried until the moisture content of the grains reached 13–14%. The grain yield per pot was the total paddy weight from each pot.

### 4.6. Experimental Design and Statistical Analyses

All pots (10 pots per treatment) were set up according to a completely randomized design (CRD). Statistix 8 (Analytical Software, Tallahassee, Florida, USA) was used to analyze the data, whereas treatment means were separated according to the least significant difference (LSD) test with 5% probability. R studio was used to generate the figures with ‘ggplot2’, ‘tidyr’, ‘dplyr’, ‘agricolae’, and ‘corrplot’ packages.

## 5. Conclusions

Both Pb and Cd, whether applied individually or in combination, induced oxidative stress, chlorophyll degradation, photosynthesis inhibition, and yield reduction in aromatic rice, with the more severe effects observed under Pb+Cd toxicity. Exogenous GABA application effectively alleviated Pb and Cd stress by modulating antioxidative defense; improving the uptake of essential mineral nutrients such as Ca, Mg, Cu, Fe, Mn, and Zn; and restricting the translocation of Pb/Cd to aboveground plant parts. Both Pb and Cd exhibited the same distributive pattern within plant parts, with the highest contents in roots, followed by stems, leaves, and grains. However, GABA-treated plants showed substantially reduced Pb and Cd accumulation in all tissues. Additionally, Pb and Cd were competitively taken up by rice plants under the combined Pb+Cd treatment. Future studies should aim to validate these findings under field conditions, assess the economic feasibility of GABA application, and explore formulation strategies for optimized delivery. Integrating GABA with other sustainable and eco-friendly agricultural practices and inputs may further enhance its potential as a viable approach for mitigating heavy metal stress in food crops.

## Figures and Tables

**Table 1:** Effect of exogenous GABA application on Pb and Cd concentrations (µg g^−1^ dry weight) in different plant parts of aromatic rice under individual and combined Pb and Cd toxicity.

	Pb				Cd			
	Roots	Stems	Leaves	Grains	Roots	Stems	Leaves	Grains
Control	28.07 ± 0.40 d	5.18 ± 0.49 d	2.96 ± 0.53 c	0.19 ± 0.01 c	4.79 ± 0.62 e	5.31 ± 0.15 d	4.26 ± 0.45 e	0.067 ± 0.00 d
GABA	27.09 ± 1.02 d	4.43 ± 0.78 d	2.52 ± 0.48 c	0.18 ± 0.01 c	4.47 ± 0.48 e	4.86 ± 0.09 d	4.82 ± 0.30 e	0.053 ± 0.01 d
Pb+GABA	1715.14 ± 54.46 c	96.77 ± 4.29 b	70.88 ± 2.82 b	1.66 ± 0.24 b	4.40 ± 1.01 e	4.28 ± 0.25 d	5.10 ± 0.29 e	0.062 ± 0.01 d
Cd+GABA	30.29 ± 0.93 d	6.96 ± 0.24 d	2.85 ± 0.38 c	0.21 ± 0.01 c	233.74 ± 3.61 bc	23.90 ± 1.37 c	13.82 ± 0.29 b	0.610 ± 0.01 b
Pb+Cd+GABA	1738.02 ± 68.67 c	74.10 ± 1.86 c	65.69 ± 2.78 b	1.63 ± 0.10 b	227.31 ± 1.06 c	20.35 ± 0.88 c	12.45 ± 0.53 c	0.488 ± 0.07 c
Pb	2559.15 ± 88.67 a	112.04 ± 5.06 a	109.54 ± 23.67 a	3.13 ± 0.34 a	31.55 ± 0.63 d	6.44 ± 1.24 d	4.20 ± 0.61 e	0.053 ± 0.00 d
Cd	13.35 ± 1.63 d	6.24 ± 0.91 d	3.69 ± 0.18 c	0.20 ± 0.02 c	263.05 ± 3.72 a	41.90 ± 2.99 a	17.92 ± 0.18 a	0.733 ± 0.04 a
Pb+Cd	1925.39 ± 25.49 b	107.93 ± 4.05 a	80.04 ± 2.19 b	2.86 ± 0.24 a	240.15 ± 5.08 b	35.23 ± 1.24 b	8.96 ± 0.15 d	0.534 ± 0.07 bc

Values are means of four replicates ± S.E. Values sharing different lowercase letter within same column differ significantly at *p* < 0.05. Control (no Pb, Cd, or GABA), GABA (1 mM), Pb (800 mg/kg of soil)+GABA, Cd (75 mg/kg of soil)+GABA, Pb+Cd+GABA, Pb, Cd, and Pb+Cd without GABA.

**Table 2:** Effect of exogenous GABA application on translocation factor (TF) of Pb and Cd from roots-to-stems-to-leaves-to-grains in aromatic rice under individual and combined Pb and Cd toxicity.

	Pb			Cd		
	Roots–Stems	Stems–Leaves	Leaves–Grains	Roots–Stems	Stems–Leaves	Leaves–Grains
Control	0.1845	0.5714	0.0642	1.1086	0.8023	0.0157
GABA	0.1635	0.5688	0.0714	1.0872	0.9918	0.0110
Pb+GABA	0.0564	0.7325	0.0234	0.9727	1.1916	0.0122
Cd+GABA	0.2298	0.4095	0.0737	0.1023	0.5782	0.0441
Pb+Cd+GABA	0.0426	0.8865	0.0248	0.0895	0.6118	0.0392
Pb	0.0438	0.9777	0.0286	0.2041	0.6522	0.0126
Cd	0.4674	0.5913	0.0542	0.1593	0.4277	0.0409
Pb+Cd	0.0561	0.7416	0.0357	0.1467	0.2543	0.0596
Means	0.1555	0.6849	0.0470	0.4838	0.6887	0.0294

Control (no Pb, Cd, or GABA), GABA (1 mM), Pb (800 mg/kg of soil)+GABA, Cd (75 mg/kg of soil)+GABA, Pb+Cd+GABA, Pb, Cd, and Pb+Cd without GABA.

**Table 3:** Description of experimental treatments.

Treatments	Description
Ck (control)	no Pb, Cd or GABA
GABA	1 mM L^−1^ GABA is applied (no Pb or Cd)
Pb+GABA	1 mM L^−1^ GABA is applied with 800 mg kg^−1^ of Pb
Cd+GABA	1 mM L^−1^ GABA is applied with 75 mg kg^−1^ of Cd
Pb+Cd+GABA	1 mM L^−1^ GABA is applied with combined Pb+Cd, i.e., 800 and 75 mg kg^−1^
Pb	only Pb (800 mg kg^−1^ of soil) is applied (no GABA)
Cd	only Cd (75 mg kg^−1^ of soil) is applied (no GABA)
Pb+Cd	both Pb and Cd are applied together (no GABA)

## Data Availability

Data are contained within the article and Supplementary Materials.

## Supplementary Materials

The following supporting information can be downloaded at: https://www.mdpi.com/article/10.3390/plants14162561/s1, File S1: Protective Role of GABA in Aromatic Rice under Lead and Cadmium Toxicity: Physiological and Biochemical Insights.
