# Supplementary material for: Protective Role of GABA in Aromatic Rice Under Lead and Cadmium Toxicity: Physiological and Biochemical Insights
Source: Plants (Basel). 2025 Aug 17;14(16):2561. doi: 10.3390/plants14162561 (PMC12389480; doi:10.3390/plants14162561)
Supplement: Supplementary file 1 [file plants-14-02561-s001.zip › plants-3715487-supplementary.pdf]

Supplementary material

Protective Role of GABA in Aromatic Rice under Lead and Cadmium Toxicity: Physiological and Biochemical Insights

Glutamine Synthetase (GS) activity:

The fresh leaves (0.2 g) were homogenized in 3 ml of Tris-HCI (pH 8.0) extraction buffer containing 1.5295g Tris, 0.1245 $MgSO_4·6H_2O$, 0.1543g DTT and 34.25g sucrose in 250ml water). The homogenate was centrifuged at 10000 rpm for 20 min, and 0.7 ml of supernatant was added to 1.6 ml of assay a solution mixture containing 3.0590g Tris, 4.9795g $MgSO_4.7H_2O$, 0.8628g glutamic acid-Na, 0.6057g cysteine 0.1920g aminopolycarboxylic acid (EGTA), and 1.3898g hydroxylamine hydrochloride in 250ml water and 0.7ml ATP (0.1210g ATP in 5ml water. The mixture was incubated for 30 min at 25°C. Subsequently, the reaction was stopped after adding 1ml of color reagents (3.3176g tri-chloro acetic acid (TCA), 10.1021g acidic $FeCl_3.6H_2O$ dissolved in water and added 5ml HCl, reached to 100ml. The samples were then centrifuged at 4,000 rpm for 15 min, and the absorbance of the supernatant was measured at 540 nm. The blank group contained 1.6ml an assay mixture containing 3.0590g Tris, 4.9795g $MgSO_4$`$7H_2O$, 0.8628g glutamic acid-Na, 0.6057g cysteine 0.1920g EGTA). The GS activity was measured as: change in absorption (ΔA×Vt)/ (Vs × FW × t), where ΔA=Change in absorbance; Vt=total volume; Vs=sample volumle; FW= fresh weight; t= reaction time.

Nitrate reductase (NR) activity:

Fresh leaves (0.25g) was precisely homogenized with 4.0 ml extraction buffer containing (0.1211g cysteine, 0.0372g EDTA-Na2 in 100ml phosphate buffer (pH 7.5), The homogenate was clarified by centrifugation at 4000 rpm for 15 min at 4°C. The supernatant was collected and assayed. Crude enzyme (0.4 ml) extract was added 1.2ml KNO_3 buffer (8.8640g $Na_2HPO_4.12H_2O$ and 0.0570g $K_2HPO_4.3H_2O$ in 1000 ml water, pH 8.7) and 0.4 ml NADH (2mg NADH in 1ml phosphate buffer (pH 7.5). The mixture was incubated for 30 min at 25°C. For control, 0.4 ml phosphate buffer was used instead of 0.4 ml NADH. Subsequently, the reaction was stopped after adding 1.0 ml of 1% 4-aminobenzene sulfonic acid and 0.2% 1-naphthylamine of each and left for 30 min for color development at 30°C. The tubes were then centrifuged at 4000 rpm for 10 min. The absorbance of the supernatant was measured at 540nm immediately. NR activity is expressed in Units h^{-1} g^{-1} FW,

which refers to amount of enzyme required to produce NO_2 in 1 h by 1 g fresh weight of plant materials.
